# Vibration-Induced Property Change in the Melting and Solidifying Process of Metallic Nanoparticles

**DOI:** 10.1186/s11671-017-2085-x

**Published:** 2017-04-26

**Authors:** Yonggang Zheng, Liquan Ding, Hongfei Ye, Zhen Chen

**Affiliations:** 1 International Research Center for Computational Mechanics, State Key Laboratory of Structural Analysis for Industrial Equipment, Department of Engineering Mechanics, Dalian University of Technology, Dalian, 116024 People’s Republic of China; 2 Department of Civil and Environmental Engineering, University of Missouri, Columbia, MO 65211 USA

**Keywords:** Additive manufacturing, Nanoparticles, Molecular dynamics

## Abstract

Tuning material properties in the 3-D printing process of metallic parts is a challenging task of current interests. Much research has been conducted to understand the effects of controlling parameters such as the particle geometry (size and shape), heating, and cooling ways on the outcome of the printing process. However, nothing has been done to explore the system vibration effect. This letter reports our findings on the vibration-induced property change in the melting and solidifying process of silver nanoparticles with the use of molecular dynamics simulation. We find that the increase of system vibration magnitude would increase the number fraction of disordered atoms, which in turn changes the nanostructure of solidified products. For a given system vibration magnitude, the number fraction of disordered atoms reaches the maximum around the system natural frequency so that the stiffness of solidified products becomes the minimum. Since this trend is not affected by the system size, the above findings reveal a feasible path toward the real-time tuning of material properties for advancing additive manufacturing.

## Background

Various 3-D printing technologies have been developed in recent years [1]. Among them, the 3-D printing methods based on the laser melting and laser sintering techniques can effectively process nanocrystalline metals [2, 3], which are playing an increasingly important role in the rapid processing and manufacturing of advanced micro-/nano-devices. Due to the small characteristic size and light weight of metallic nanoparticles, however, the sintering behavior and mechanism of these particles have fundamental differences from those of the conventional metallic particles. In addition, they can also be influenced by various environmental factors (such as heating and cooling rates, system vibration), which will eventually alter the physico-mechanical properties of the final sintered products. Thus, studies on the sintering behavior and mechanism of metallic nanoparticles are of great scientific significance and practical value.

To understand the sintering, including the melting and solidifying, process of nanoparticles, extensive experimental studies have been conducted worldwide. For examples, Link et al. found experimentally the morphological changes of spheroidal gold nanoparticles or nanorods into nanospheres via melting at moderate laser energies [4, 5]. Kim and Jang observed that picosecond laser pulses can induce the nanowelding of gold nanoparticles and the formation of single-phased nanocontact, which is useful for the fabrication of ohmic contact [6]. Ko et al. demonstrated that the low-temperature metal deposition as well as high resolution pattern can be achieved via the laser sintering of inkjet-printed metal nanoparticles, which overcomes the resolution limitation of the current inkjet direct writing processes [2]. Moreover, molecular dynamics (MD) simulations have also been widely used to investigate the sintering behavior of various metallic nanoparticles. Raut et al. studied the sintering of aluminum nanoparticles by MD simulations, and they found that the increase of the particle size may slow down the sintering kinetics [7]. Zeng et al. performed MD simulations to study the surface energies, grain boundary mobility, and sintering of copper and gold nanoparticle arrays at different temperatures, and their results show unexpected contributions from plastic deformation, mechanical rotation, amorphization, and ultrarapid diffusion effects [8]. Koparde and Cummings demonstrated that the dipole-dipole interaction between sintering titanium dioxide nanoparticles plays a very important role at the temperature far from the melting point [9]. Wang et al. studied the ultrafast laser interaction with free gold nanoparticles using the MD and two-temperature models, and they found that a nonhomogeneous surface premelting mechanism is dominant at a low laser intensity and the appearance of a contiguous surface liquid layer is size dependent [10]. Jiang et al. studied the necking growth behavior in laser sintering of hollow silver nanoparticles under different heating rates, and they revealed that the melting temperature vs. heating rate shows an inverse trend in all the hollow nanoparticle pairs at an ultrahigh heating rate as compared to that in the solid particle cases [11]. Moreover, Arcidiacono et al. demonstrated the reliability of MD simulations for the sintering of metallic nanoparticles based on the results for the necking growth obtained from both simulations and theoretical predictions [12]. Although many efforts have been made to understand the sintering behavior of metallic nanoparticles, the previous studies were mainly focused on the melting process. To the authors’ knowledge, little has been done to explore the environmental effects on the sintering behavior and corresponding mechanical features of the final products. However, the environmental factors, such as the vibration of the experimental platform, might greatly affect the melting and solidifying process and eventually the microstructure of the final products. Furthermore, it is the physico-mechanical properties of the sintered products that are of practical importance.

In this paper, the environmental effects on the melting and solidifying behaviors and corresponding material properties of sintered products obtained from silver nanoparticles are investigated using MD simulations. The influences of the temperature conditions (including the final heating temperature and heating rate), vibration factors (including amplitude and frequency), and the size of nanoparticles are reported.

## Methods

**Fig. 1:**
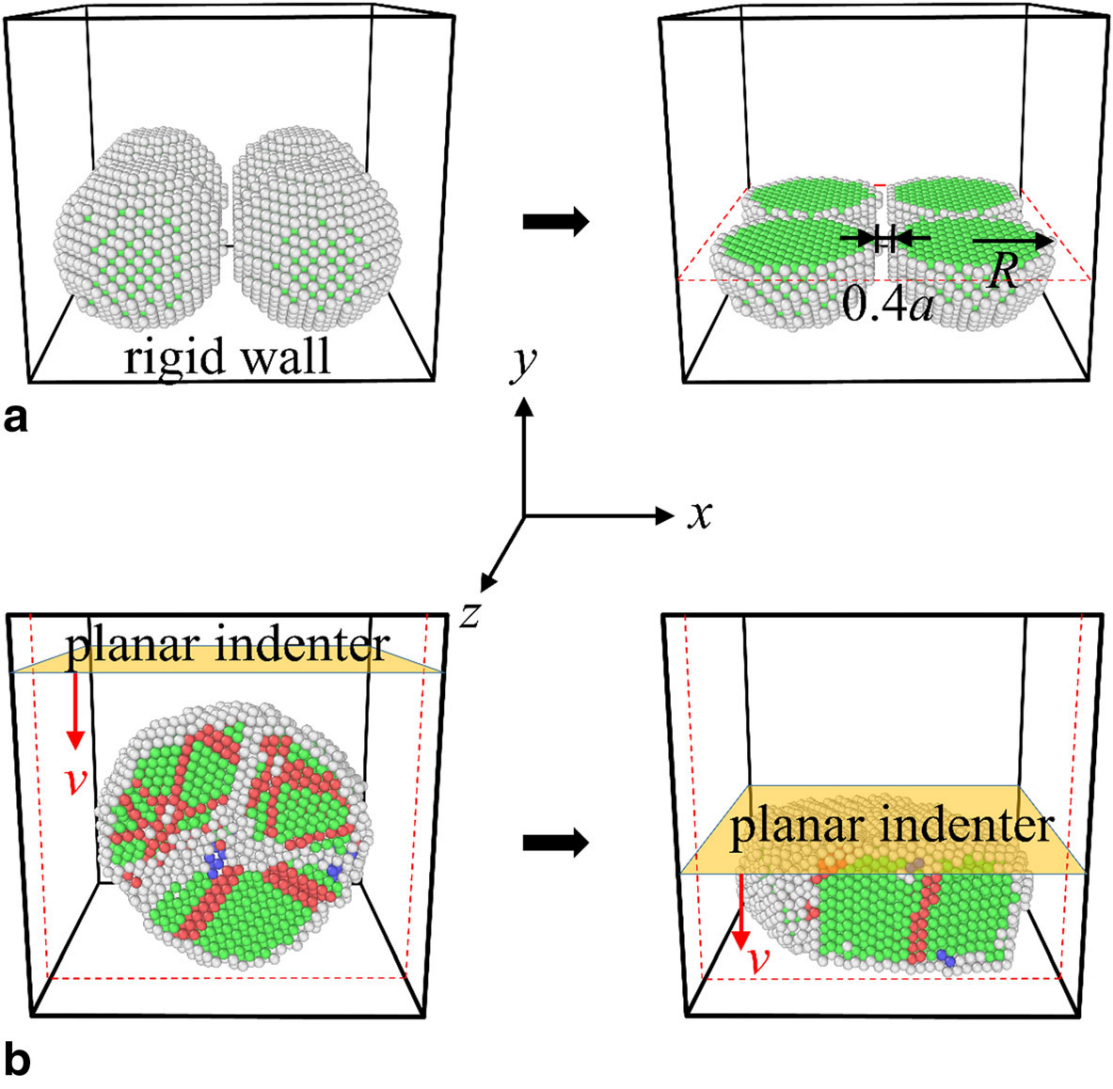
Schematic plots of the simulated system. **a** Schematic plots of the initial system and the corresponding sectional view (cut by the plane represented by *red dashed lines*), in which the geometrical sizes are denoted. **b** Schematic plots (sectional view) of the compressed sintered product at different times, in which the planar indenter moves downwards with a constant velocity *v*. Here, the atoms in *green, red*, and *blue* are those in local FCC, hexagonal-close-packing (HCP), and body-centered-cubic (BCC) lattices, respectively, while disordered atoms in *gray* represent surfaces and dislocation cores. The planes bounded by *solid lines* represent six rigid walls

The simulated system for this study contains four silver nanoparticles, and the corresponding geometries are shown schematically in Fig. 1a. The four nanoparticles are created as solid spheres with a prescribed radius *R* extracted from a bulk face-centered-cubic (FCC) silver crystal with the [100], [010], and [001] crystallographic directions, which coincide with the *x*-, *y*-, and *z*-axes, respectively. Four nanoparticles have the same size and are bounded by six rigid walls, which mimic a portion of the deposited spherical nanopowders at the surface layer of a system in a real sintering experiment. To study the particle size effect on the sintering behavior and mechanical properties of the final product, the radius *R* is varied as 5*a* and 10*a* with *a* being the lattice constant of silver crystal (i.e., *a* = 4.09 Å), respectively. The centroids of the four particles have the same *y* coordinate, and the initial gap between these nanoparticles is set to be ~ 0.4*a*.

The interactions between silver atoms are described by the embedded atom method [13]. The simulations are conducted by using the open-source large-scale MD simulator LAMMPS [14]. The nanoparticles are initially relaxed at 0 K separately using the conjugated gradient method and then are put together to form the simulated systems (as shown in Fig. 1a). During the laser sintering process, the system is first thermally equilibrated at 298 K using the Nosé-Hoover thermostat [15, 16] for 1 ns to mimic the solid-state sintering. The system temperature is then increased linearly via the velocity rescaling method with a constant heating rate to model the laser sintering process. After reaching a specified temperature, the system is equilibrated at that temperature for another 1 ns. Subsequently, the system temperature is decreased linearly to 298 K with a cooling rate that is the same as the heating rate. Finally, the cooled specimen is equilibrated at 298 K for 1 more nanosecond to obtain the final sintered product. During the laser sintering process, the effects of the heating/cooling rate and system vibration on the sintering behavior are explored. To evaluate the mechanical properties of the sintered product, compressive tests with a planar indenter are carried out, as shown in Fig. 1b. For all simulations, a time step of 2 fs is used. The common neighbor analysis method [17, 18] is used to determine the local crystalline order of silver atoms in order to identify the dislocation core, stacking fault, deformation twin, and nanostructure evolution [19, 20] which can be visualized by OVITO [21].

## Results and Discussion

### Melting and Solidifying Process of Silver Nanoparticles

**Fig. 2:**
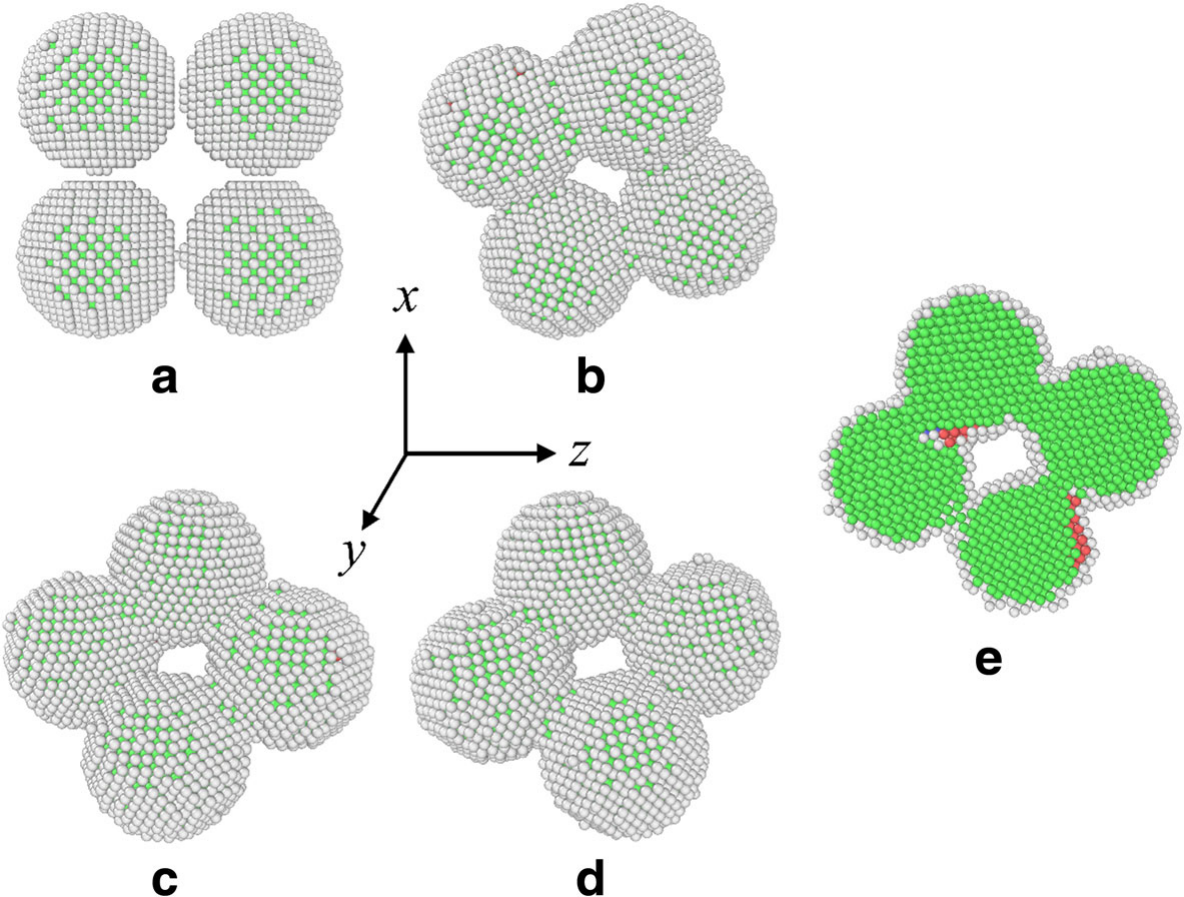
Nanostructure evolution and rotation of silver nanoparticles during solid state sintering at 298 K. **a** 0 ns. **b** 0.34 ns. **c** 0.68 ns. **d** 1.00 ns. **e** Sectional view at 1.00 ns

To investigate the melting and solidifying process of silver nanoparticles and also to explore the mechanical properties of the sintered product, systems that contain four silver nanoparticles are prepared and compressive tests are carried out (see Fig. 1). During the equilibrium process of the as-fabricated four silver nanoparticles at the room temperature, it is found that solid-state sintering occurs. As shown in Fig. 2, the initially separated nanoparticles approach to each other, accompanied with rotation and slightly plastic deformation, which are consistent with previous studies [8, 11]. Once the particles contact with the adjacent ones, dumbbell-like necks can be formed rapidly between two particles. The neck widths approach constant values after a short period and are almost the same since the four particles have the same radius.

**Fig. 3:**
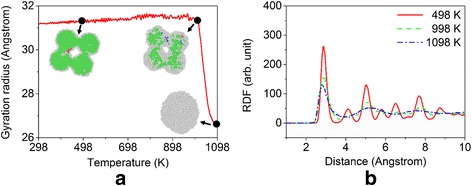
Evolution of the gyration radius and radial distribution function during the heating process. **a** Gyration radius as a function of temperature during the heating process, in which the *insets* show the corresponding sectional view of the nanostructures at 498, 998, and 1098 K. **b** The corresponding radial distribution functions (RDF) of the system at 498, 998, and 1098 K

During the heating stage in the laser sintering process, the system temperature is linearly increased to 1098 K via the velocity rescaling approach. Figure 3a illustrates the nanostructure evolution of the system and the gyration radius as a function of temperature. Here, the gyration radius of the system is defined as the root mean square distance of atoms of silver nanoparticles measured from their centers of mass that is [11]

$$ {R}_{\mathrm{g}}(t)=\sqrt{\frac{1}{M}{\displaystyle \sum_{i=1}^N}{m}_i{\left[{\boldsymbol{r}}_i(t)-{\boldsymbol{r}}_{\mathrm{cm}}(t)\right]}^2} $$

where *N* is the total number of atoms in the system, *M* is the total mass of the system, *t* represents time, ***r***_*i*_ is the position of the *i*th atom, and ***r***_cm_ is the center-of-mass position of all nanoparticles. It can be seen that the system keeps relatively stable and the overall shape changes a little when the temperature is less than ~998 K. With the further increase of temperature, however, the nanoparticles expand slightly and thus the gyration radius increases slightly. After increasing to 998 K, the surface portions of nanoparticles begin to melt and fuse with each other, accompanied by the disappearing of the dumbbell-like necks. The gyration radius decreases drastically at this stage. When the temperature approaches to 1098 K, four nanoparticles completely melt and form a single liquid spherical particle to reduce the free surface area and thus reduce the total energy of the system. The gyration radius keeps relatively stable after that. The radial distribution functions at different temperatures are shown in Fig. 3b, which further confirm the melting of the nanoparticles from crystalline phase at a high temperature.

**Fig. 4:**
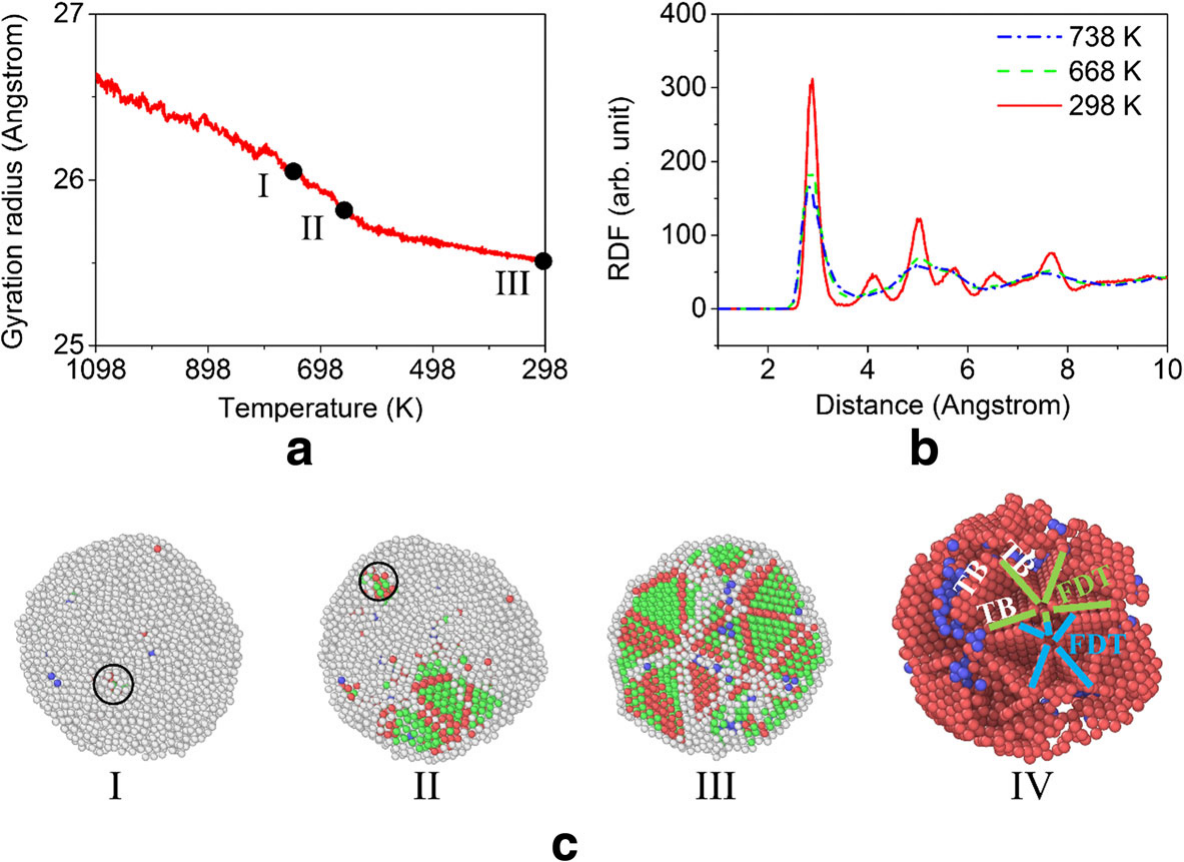
Evolution of the gyration radius, radial distribution function, and the nanostructure during the cooling process. **a** Gyration radius as a function of temperature during the cooling process and **b** the corresponding radial distribution functions (RDF) of the system at 738, 668, and 298 K. **c** Sectional view of the nanostructures at *I* 738 K, *II* 668 K, and *III* 298 K, and *IV* the nanostructure of the final sintered product, in which circles indicate the nucleation cores. In addition, disordered and FCC atoms are removed, and two fivefold deformation twins (FDT, five short lines with the same color are used to represent an FDT) and several twin boundaries (TB) are marked in (*IV*) for clarity

Figure 4a, b shows the gyration radius as a function of temperature and the radial distribution function at several representative times during the cooling process. It can be seen that the gyration radius decreases with the decrease of temperature, which is mainly attributed to the solidification and crystallization of the system. Thus the radial distribution function approaches the solid-like feature with the decrease of the temperature. Moreover, from the nanostructure evolution as shown in Fig. 4c (I–III), we can see that the liquid sphere-like particle gradually crystallizes and this process consists of two main phases, i.e., the nucleation phase and crystal growth phase. Several nucleation cores form in the interior of the particle (see the circular areas in the nanostructure pictures as shown in Fig. 4c (I and II)) and grow gradually. The existence of only a limited number of nucleation sites observed in our simulations can be attributed to the relative small system size (about 100 – 1000 nm^3^ for different systems) and short quenching time (about 1 – 20 ns for the systems under different cooling rates), which is reasonable according to the analyses in [22]. After being equilibrated at the room temperature, the final products are mostly polycrystalline materials with grain-like portions separated by grain boundaries composed of amorphous atoms. Moreover, it can be found from Fig. 4c (IV) that there are many multiple twins, especially the fivefold twins, in the final products, which can be attributed to the successive formation of twin hexagonal-close-packing (HCP) planes during the layer-by-layer growth of FCC stacking block [23, 24]. Table 1 presents the number fraction of atoms with different local crystalline orders at several representative cooling temperatures. It can be seen that in the final sintered product most of the atoms are disordered due to the presence of surface and abundant grain boundaries. The number fraction of atoms in local FCC is comparable to that of atoms in local HCP, which further implies the existence of extensive twin boundaries in the sintered sample.

**Table 1:** Number fraction of atoms with different local crystalline orders at 1098, 738, 668, and 298 K during the cooling process

Temperature (K)	1098	738	668	298
Disordered (%)	100.0	99.4	86.3	44.8
FCC (%)	0.0	0.1	7.1	30.2
HCP (%)	0.0	0.4	5.8	22.6
BCC (%)	0.0	0.1	0.8	2.4

**Fig. 5:**
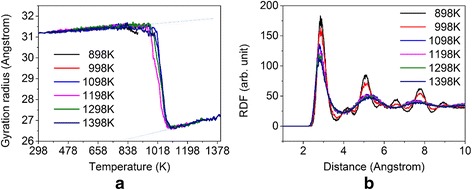
Influence of the maximum heating temperature. **a** Gyration radius as a function of temperature during the heating process. **b** The final radial distribution function (RDF) of the systems with different maximum temperatures. *Dashed lines* in (**a**) indicate the slope of the curve at different heating stages

Actually, the melting and solidifying process may be influenced by many factors, including the highest heating temperature, the heating/cooling rate, and the system vibration. We first investigate the effect of the highest temperature during the heating process on the sintering behavior, and the results are shown in Fig. 5. It is found that the highest temperature has certain influences on the fusion of nanoparticles. With the increase of the highest temperature, nanoparticles gradually melt and fuse completely. When the highest temperature exceeds 1098 K, the extent of fusion of nanoparticles is almost the same. As mentioned before, the gyration radius of system gradually increases when the temperature is less than ~998 K due to the thermal expansion of the solid particles, and the gyration radius decreases rapidly when the temperature increases from 998 to 1098 K due to the complete melting of nanoparticles. However, the gyration radius of the liquid sphere particle increases again when the temperature exceeds 1098 K due to the thermal expansion of the liquid-like particle. The slope of the gyration radius vs. temperature for the liquid-like particle is slightly larger than that for the solid nanoparticle, which implies that the former has a slightly larger thermal expansion coefficient.

**Fig. 6:**
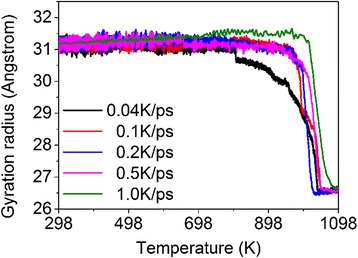
Gyration radius as a function of temperature during the heating process with different heating rates

We then examine the effect of heating/cooling rate on the sintering behavior of silver nanoparticles. Here the maximum temperature is set to be 1098 K for all cases with different heating rates, including 0.04, 0.1, 0.2, 0.5 and 1 K/ps. Figure 6 shows the gyration radius as a function of temperature under different heating rates. It can be seen that with the increase in heating rate, the initial fusion time generally increases, that is, the fusion of nanoparticles exhibits hysteresis for high heating rate. However, the magnitude of the slope of gyration radius vs. temperature in the range of ~950 to ~1050 K becomes large for high heating rate, which implies that the higher the heating rate, the faster the fusion. Moreover, the degrees of fusion at the final time become similar for each case, and the radial distribution functions are almost the same (not shown here).

**Fig. 7:**
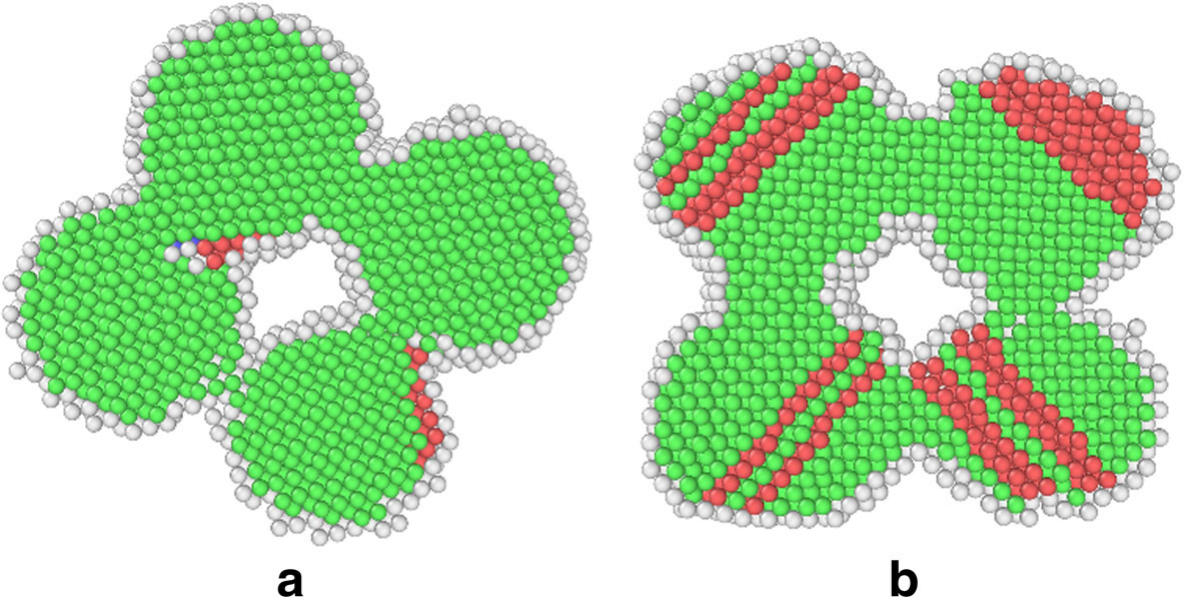
Nanostructure of the solid state sintered silver nanoparticles. **a** Without system vibration. **b** With system vibration

Finally, we explore the vibration effect of the experimental platform on the sintering behavior of silver nanoparticles, with the above MD simulation system. For the purpose of demonstration, a sinusoidal transverse vibration is considered in the present work, and the vibration direction coincides with the *z* axis with an amplitude of 3*a* and a period of 9 ps as a reference value. The final temperature is set to be 1098 K and the heating/cooling rate is 1 K/ps. The nanostructures for the systems with and without vibration after the solid-state sintering at the room temperature are shown in Fig. 7. It can be seen that the interior of the system without system vibration keeps a nearly perfect FCC lattice, while the system with vibration contains many atoms with HCP lattice (number fraction is about 20%) due to the plastic deformation induced by the impact between different nanoparticles or between nanoparticles and rigid walls.

**Fig. 8:**
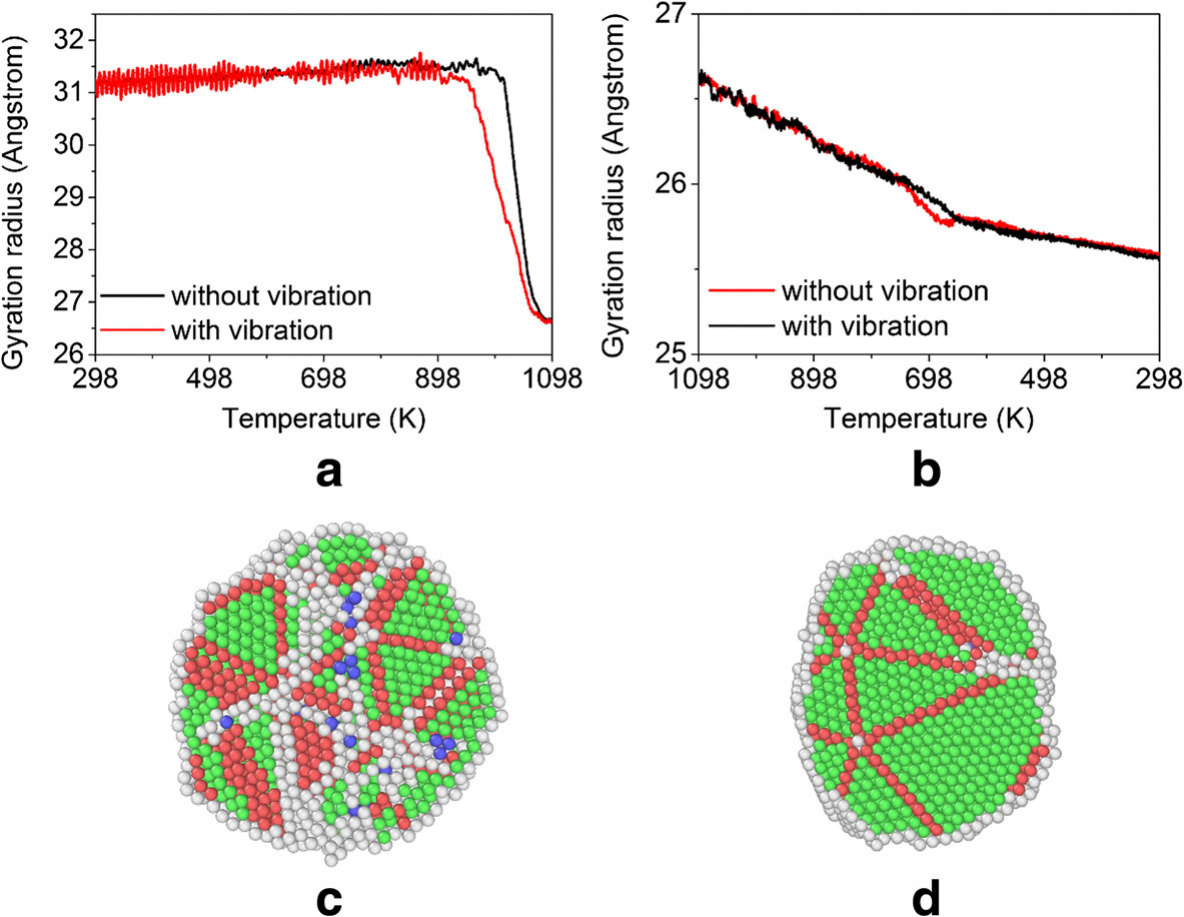
Influence of the environmental vibration. **a**, **b** Gyration radius as a function of temperature during the heating and cooling processes with and without system vibration. **c**, **d** Nanostructures of the sintered silver nanoparticles without vibration and with vibration, respectively

The gyration radii as a function of temperature during the heating processes are shown in Fig. 8a for the two systems with and without system vibration. We can observe that the two curves almost coincide with each other at the earlier laser sintering stage except that relatively large fluctuations appear for the system with vibration. However, the nanoparticles with system vibration tend to melt earlier than those without system vibration. Moreover, the final degrees of fusion of nanoparticles are almost the same for both cases. During the cooling process, the gyration radii as a function of temperature for the systems with and without vibration are shown in Fig. 8b. It can be seen that the overall trends for both curves are similar. However, the gyration radius of the system with vibration is slightly smaller than that of the system without vibration, which indicates that the fusion of nanoparticles in the formal system is much better than that in the latter. This behavior is also found in the system with different final sintering temperatures (i.e., 1198 and 1298 K). The nanostructures after cooling are shown in Fig. 8c, d. It can be seen that there are less disordered atoms in the system with vibration, which implies that the vibration could rearrange the atoms to a more compact fashion and further confirms the better fusion of nanoparticles in this system. However, the outcome of this phenomenon is dependent on vibration frequency and amplitude, as illustrated in the next subsection.

### Mechanical Properties of Sintered Nanoparticles

**Fig. 9:**
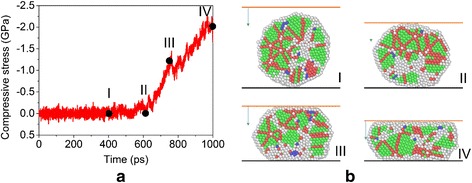
Compressive test of sintered nanoparticles. **a** Compressive stress as a function of time for the final sintered particle. **b** Nanostructures at *I* 400 ps, *II* 610 ps, *III* 760 ps, and *IV* 1000 ps, in which *solid lines* represent rigid wall (*black*) and planar indenter (*red*) and the *arrow* indicates the moving direction of the indenter

To evaluate the mechanical properties of the final sintered products, a series of compressive tests are carried out. As shown in Fig. 1b, a planar indenter is initially placed above the products and then move toward the products with a constant velocity of 0.06 Å/ps. The compressive stress vs. time is plotted in Fig. 9a, and the corresponding nanostructures at several representative times are shown in Fig. 9b (I–IV). It can be seen that, at the initial stage, the compressive stress is about 0 GPa with small fluctuations. Actually, the planar indenter does not touch the particle in this stage (see Fig. 9b (I)). When the indenter contacts with the particle, the compressive stress increases gradually with the increase of the indentation depth. The shape of the product changes from sphere-like to drum-like. The plastic deformation of the particle is mainly accommodated by grain boundary and partial dislocation activities.

**Fig. 10:**
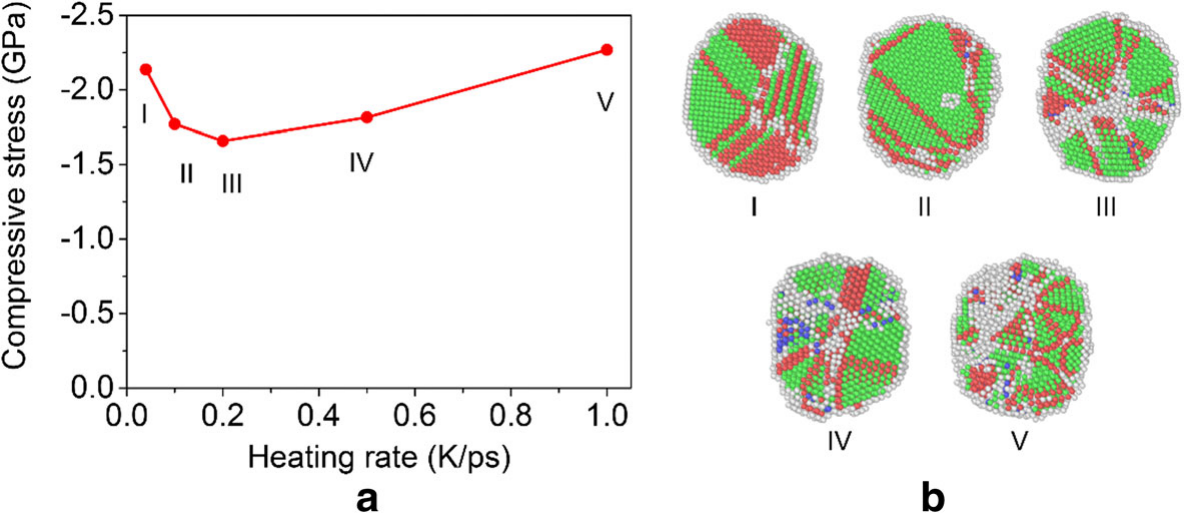
Influence of the heating rate on the mechanical properties of sintered nanoparticles. **a** Final compressive stress as a function of heating rate for the sintered particles without considering the system vibration. **b** Corresponding nanostructures of the sintered nanoparticles under different heating rates

To facilitate the evaluation of the mechanical properties of the product sintered under different conditions, we measure the compressive stress corresponding to the same final indentation depth for different cases (i.e., 2.5 nm after contact). The final compressive stress as a function of the heating/cooling rate is shown in Fig. 10a. It can be seen that the magnitude of the stress decreases with the increase of the heating/cooling rate when the rate is low, while it increases with the rate when the rate is high. By examining the number fraction of atoms with different crystalline orders after the cooling of the systems (i.e., the final sintered samples), see Table 2 and Fig. 10b, it can be seen that the system first tends to become more compact as the increase of the heating/cooling rate and the magnitude of the final compressive stress decreases, which implies that the system ductility increases (i.e., the system has a much lower compressive stress at the same indentation depth). By further increasing the heating rate, the system becomes more disordered and has an increased compressive stress.

**Table 2:** Number fraction of atoms with different local crystalline orders in the products sintered under different heating rates

Heating rate (K/ps)	0.04	0.1	0.2	0.5	1.0
Disordered (%)	27.5	27.5	35.6	39.8	44.8
FCC (%)	32.5	45.6	40.1	37.0	30.2
HCP (%)	40.0	26.8	23.7	20.3	22.6
BCC (%)	0.0	0.1	0.6	2.9	2.4

**Fig. 11:**
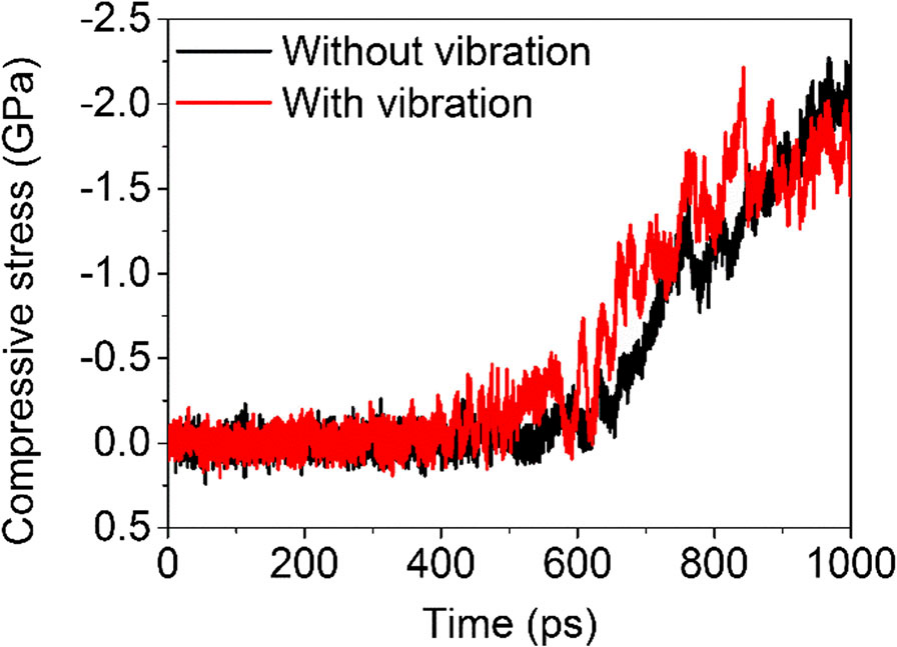
Compressive stress as a function of time for the finial sintered particles with and without system vibration

The system vibration also affects the mechanical properties of sintered products. As can be observed from Fig. 11, the stress vs. time curve for the system with vibration exhibits much larger fluctuations but a stiffer response than that for the system without vibration.

**Fig. 12:**
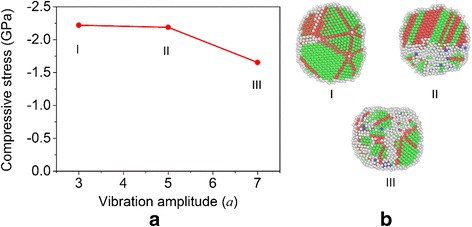
Influence of the vibration amplitude on the mechanical properties of sintered nanoparticles. **a** Final compressive stress as a function of vibration amplitude for the sintered particles. **b** Nanostructures of the nanoparticles sintered under different vibration amplitudes

To further explore the system vibration effect on the mechanical properties of sintered nanoparticles, compressive tests are performed for the systems produced at different vibration amplitudes and frequencies. Figure 12a shows the final compressive stress as a function of vibration amplitude for the nanoparticles sintered under the same frequency of ~0.111 THz. It can be seen that the stress slightly decreases with the increase of the vibration amplitude while it decreases rapidly to a much small value when the amplitude becomes large. The transition from a small change to big change in compressive stress is accompanied by the transition from an increase to decrease in the number fraction of HCP atoms with a monotonic increase in the number fraction of disordered atoms, as can be seen from Table 3 and Fig. 12b. When the vibration amplitude becomes large, the rapid decrease of the compressive stress appears to be mainly attributed to the much disordered state in this case as compared to others. The trend of the compressive stress vs. nanostructure is different from that shown in Fig. 10, which might be induced by the vibration of the system and calls further study in the future.

**Table 3:** Number fraction of atoms with different local crystalline orders in the products sintered under different vibration amplitudes

Amplitude	3*a*	5*a*	7*a*
Disordered (%)	27.3	38.1	47.1
FCC (%)	54.9	34.0	32.6
HCP (%)	17.7	26.7	19.0
BCC (%)	0.1	1.2	1.3

**Fig. 13:**
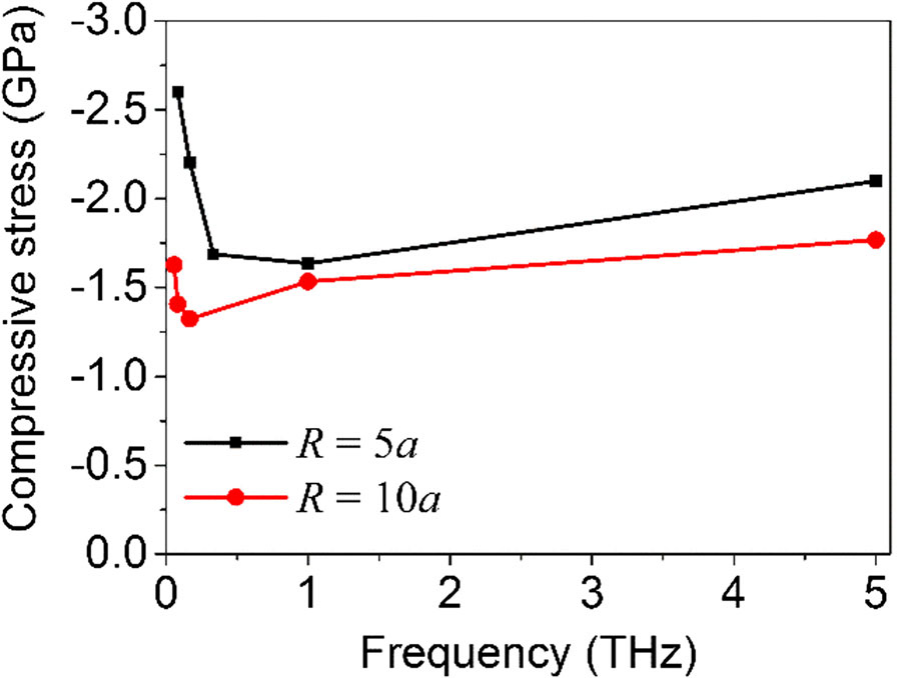
Final compressive stress as a function of vibration frequency for the sintered nanoparticles

Figure 13 shows the final compressive stress as a function of system vibration frequency for the particles sintered under the same amplitude of 3*a*. It can be seen that the final stress decreases first with the increase of the vibration frequency and then increases with further increasing the frequency. The similar tendency exists for the systems with different initial radii (two groups of systems with different initial particle radii are considered here). In other words, the frequency dependency appears to be insensitive to the system size. The minimum compressive stress (see Fig. 13) and corresponding number fraction of disordered atoms in the system (see Tables 4 and 5) occur when the system vibration frequency is ~1 THz and ~0.167 THz for the systems with an initial particle radius of 5 and 10*a*, respectively, which are around the natural vibration frequency of the corresponding sintered nanoparticle. This implies that, for a given external vibration magnitude, the number fraction of disordered atoms reaches the maximum around the system natural frequency so that the stiffness of sintered products becomes the minimum.

**Table 4:** Number fraction of atoms with different local crystalline orders in the products sintered under different vibration frequencies (*R* = 5*a*)

Frequency (THz)	0.083	0.167	0.333	1	5
Disordered (%)	31.2	31.7	37.8	39.2	24.3
FCC (%)	47.6	46.0	38.1	33.0	23.3
HCP (%)	19.7	0.5	22.0	26.2	52.4
BCC (%)	1.5	0.0	2.2	1.5	0.0

**Table 5:** Number fraction of atoms with different local crystalline orders in the products sintered under different vibration frequencies (*R* = 10*a*)

Frequency (THz)	0.056	0.083	0.167	1	5
Disordered (%)	40.3	41.4	46.4	41.8	39.1
FCC (%)	37.3	33.2	32.5	35.0	32.7
HCP (%)	20.1	22.2	17.2	20.2	25.7
BCC (%)	2.4	3.2	3.9	3.0	2.5

## Conclusions

In this work, atomistic simulations have been conducted to investigate the melting and solidifying behaviors of silver nanoparticles and the mechanical properties of the final products sintered under different environments. The influences of the heating/cooling rate and the vibration amplitude and frequency have been explored. Major findings are summarized as follows: (1) Solid-state sintering occurs when nanoparticles are relaxed at the room temperature and the adjacent particles are close to each other, which is accompanied by rotation and eventually forming dumbbell-like solid products. With the increase of temperature, dumbbell-like products begin to melt. When the maximum temperature exceeds 1098 K, silver nanoparticles completely melt and form a single liquid nanoparticle. In the cooling process, the liquid particle gradually solidifies and this process consists of two main phases, i.e., the nucleation phase and crystal growth phase. The final products equilibrated at the room temperature are mostly polycrystalline materials with grains separated by grain boundaries, stacking fault, twin boundary, etc. (2) The higher the heating rate, the later the initial fusion of nanoparticles and the lesser the time of fusion stage. (3) External vibration could affect the evolution of nanostructures of the sintered products. The number fraction of disordered atoms in the sintered products increases monotonically with the increase of the vibration amplitude for a specific frequency, and reaches the maximum as the vibrational frequency approaches the natural frequency of the system for a given amplitude. As a result, the material properties of sintered products depend on both vibration frequency and amplitude. (4) The plastic deformation of sintered products under compression is mainly dominated by grain boundary and partial dislocation activities. (5) The above phenomena appear to be independent of the simulation system size. Although the present results provide many insights to understand the sintering behaviors of more realistic multiple-particle systems, it should be noted that only limited ranges of particle size and number, heating/cooling rate, vibration frequency, and amplitude have been considered in the present work. In the future, multiscale investigation is required to design a feasible path toward the real-time tuning of material properties in the 3-D printing process of metallic parts.
